# Retraction Note: Premarital Sexual Behavior among male college students of Kathmandu, Nepal

**DOI:** 10.1186/s12889-023-16374-4

**Published:** 2023-07-28

**Authors:** Ramesh Adhikari, Jyotsna Tamang

**Affiliations:** 1 Geography and Population Department, Mahendra Ratna Campus, Tribhuvan University, Kathmandu, Nepal; 2 Center for Research on Environment Health and Population Activities (CREHPA), Kathmandu, Nepal

**Retraction Note:** ***BMC Public Health*** **9, 241 (2009)**

10.1186/1471-2458-9-241

The Editor has retracted this article due to the author’s inability to provide documentation of approval from an ethics committee.

Ramesh Adhikari does not agree with this retraction. Jyotsna Tamang has not responded to correspondence from the Editor about this retraction.

